# On-Chip Fabrication of Cell-Attached Microstructures using Photo-Cross-Linkable Biodegradable Hydrogel

**DOI:** 10.3390/jfb11010018

**Published:** 2020-03-15

**Authors:** Masaru Takeuchi, Taro Kozuka, Eunhye Kim, Akihiko Ichikawa, Yasuhisa Hasegawa, Qiang Huang, Toshio Fukuda

**Affiliations:** 1 Department of Micro-Nano Mechanical Science and Engineering, Nagoya University, Nagoya 4648603, Japan; yasuhisa.hasegawa@mae.nagoya-u.ac.jp; 2 Department of Mechatronics Engineering, Meijo University, Nagoya 4688502, Japan; 140447024@ccmailg.meijo-u.ac.jp (T.K.); kim@meijo-u.ac.jp (E.K.); ichikawa@meijo-u.ac.jp (A.I.); qhuang@bit.edu.cn (Q.H.); tofukuda@meijo-u.ac.jp (T.F.); 3 Intelligent Robotics Institute, School of Mechatronical Engineering, Beijing Institute of Technology, Beijing 100081, China

**Keywords:** microfabrication, cell culture, microfluidics, biodegradable material, photo cross-linkable material

## Abstract

We developed a procedure for fabricating movable biological cell structures using biodegradable materials on a microfluidic chip. A photo-cross-linkable biodegradable hydrogel gelatin methacrylate (GelMA) was used to fabricate arbitrary microstructure shapes under a microscope using patterned ultraviolet light. The GelMA microstructures were movable inside the microfluidic channel after applying a hydrophobic coating material. The fabricated microstructures were self-assembled inside the microfluidic chip using our method of fluid forcing. The synthesis procedure of GelMA was optimized by changing the dialysis temperature, which kept the GelMA at a suitable pH for cell culture. RLC-18 rat liver cells (Riken BioResource Research Center, Tsukuba, Japan) were cultured inside the GelMA and on the GelMA microstructures to check cell growth. The cells were then stretched for 1 day in the cell culture and grew well on the GelMA microstructures. However, they did not grow well inside the GelMA microstructures. The GelMA microstructures were partially dissolved after 4 days of cell culture because of their biodegradability after the cells were placed on the microstructures. The results indicated that the proposed procedure used to fabricate cell structures using GelMA can be used as a building block to assemble three-dimensional tissue-like cell structures in vitro inside microfluidic devices.

## 1. Introduction

Recently, cell-assembly technologies for tissue engineering and organs-on-a-chip have gained increasing attention [1,2,3] with the development of pluripotent stem cells [4,5]. Although artificial organs and related transplant technologies have improved, the side effects arising from defective artificial organs require further examination. Tissue engineering technology has matured rapidly recently [6,7,8], enabling organs to be artificially grown from stem cells.

Various assemblies of three-dimensional (3D) cell structures have been proposed in the literature. For example, two-dimensional (2D) cell sheets can be used to create thicker cell structures by picking them up and placing them layer by layer [9,10]. However, thicker structures are difficult to achieve this way, because the thickness can only be several tens of microns. To achieve 3D cell structures of larger size, robotics technologies have been applied. For example, a bio-printing method based on inkjet technologies was recently developed [11,12,13,14,15] to increase the fabrication speed and the strength of cell structures. Hydrogels (e.g., alginates) have been employed to eject and encapsulate cells inside 3D structures. However, fabrication speeds are limited, especially when the fabricated structure becomes larger, because the 3D printer must fabricate 3D structures from one-dimensional dots (i.e., droplets).

Fluidic assembly is a suitable approach for the high-throughput assembly of microstructures, including biological objects [16]. For example, complex 3D structures have been assembled by cell-encapsulated blocks [17]. Tubular structures made of biological cells have been assembled using a self-assembly process [18]. Different 3D microstructures have been also assembled inside microfluidic devices by self-assembly process. Hence, micro-assembly inside a microfluidic device shows great potential for high-throughput self-assembly of 3D cellular structures [19,20].

A photo-cross-linkable resin was employed to encapsulate cells for 3D structures using self-assembly process inside a microchannel [1,21]. In these studies, 3D structures were fabricated from 2D cell structures, revealing the potential to achieve higher assembly efficiencies compared with bio-printing. However, non-biodegradable materials were generally employed for a photo-cross-linkable resin. For this study, we developed a procedure for fabricating movable biological cell structures using a biodegradable material in a microfluidic chip, in which cells are cultured on microstructures to achieve 2D cell structures. The fabricated microstructures are movable and can be used as building blocks for 3D assembly of in vitro cell structures.

The main contributions of this study are the achievement of cell cultures on biodegradable hydrogel microstructures and the assembly demonstration of movable hydrogel microstructures inside a microfluidic device. The fabricated microstructures with cells can be used as building blocks for 3D tissue-like cell structures. The assembly of microstructures can be conducted inside the microfluidic chip using a self-assembly process described previously [1]. A demonstration of assembling GelMA microstructures inside the two-layered microchannel was conducted. The fabrication and assembly procedures require microrobotics technologies of microfabrication and micromanipulation.

## 2. Materials and Methods

### 2.1. Preparation of Biodegradable Microstructures

**Figure 1:**
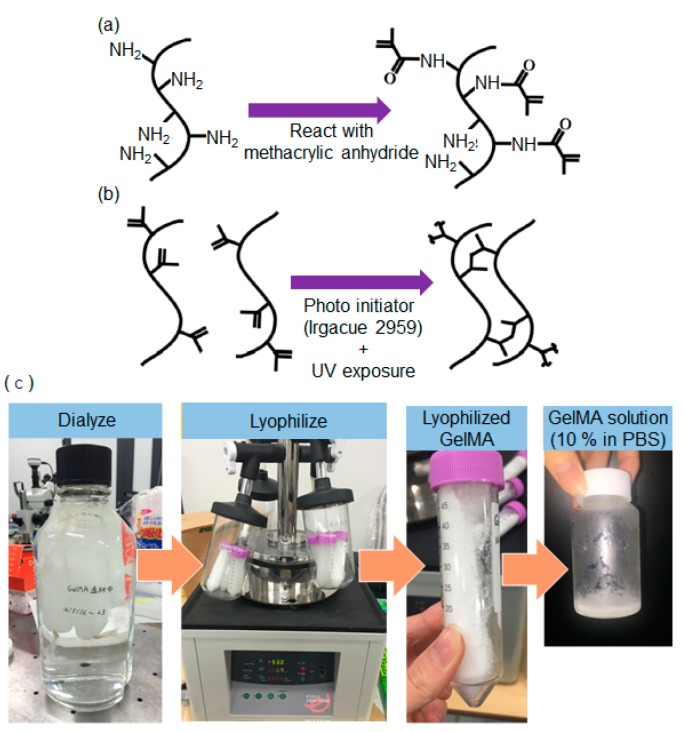
GelMA synthesis: (**a**) chemical reaction with methacrylic anhydride; (**b**) chemical reaction during UV exposure; (**c**) procedure of GelMA synthesis.

The GelMA was synthesized as follows [22,23,24,25]. First, type-A porcine skin gelatin was added at 10% w/v to phosphate-buffered saline (PBS) and heated at 50 °C for 1 h with stirring. Methacrylic anhydride (Sigma-Aldrich Japan, Tokyo, Japan) was then added (7.5% v/v) to the gelatin solution at around 60 °C while stirring, allowing it to react for 2 h. In synthesized methacrylated gelatin, gelatin macromers containing primary amino groups were reacted using methacrylic anhydride to add methacrylate pendant groups, as shown in Figure 1a. The samples were then dialyzed using 12–14 kDa cutoff dialysis tubing (Spectra/Por(R), Repligen, Waltham, MA, USA) in pure water for 1 week. Finally, the solution was frozen overnight (−80 °C), lyophilized for 1 week in a freeze-dryer (FDU-1200, Eyela, Tokyo, Japan), and stored at −80 °C until further use. The lyophilized GelMA was a white porous foam, as shown in Figure 1c. The lyophilized GelMA was dissolved in PBS or culture medium to fabricate GelMA microstructures.

### 2.2. Cell Culture in GelMA Microstructures

The GelMA solution was exposed to UV light to fabricate GelMA microstructures, and the light was measured. In our experiment, the UV power source (U-HGLGPS, Olympus, Tokyo, Japan) had a wavelength from 350 nm to 750 nm, and the output power could be changed to six different percentages: 3%, 6%, 12%, 25%, 50%, and 100%. To check the fabrication conditions, the UV power at the fabrication area of microstructure was measured. The UV light from the UV power source was exposed to the fabrication area using the 60× objective lens.

**Figure 2:**
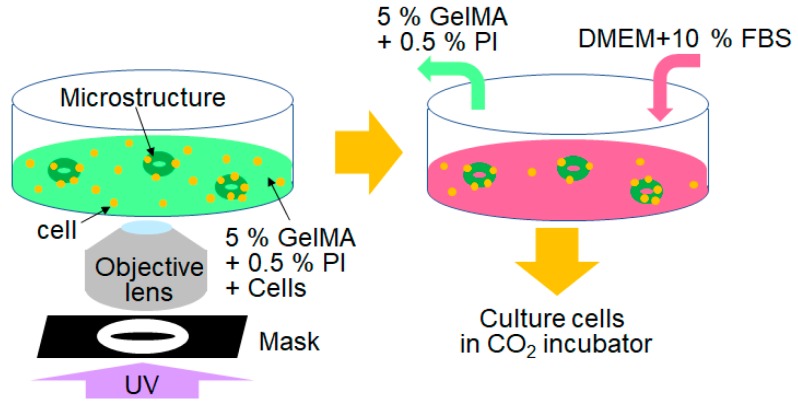
Fabrication of cell-embedded microstructures using GelMA.

The cell culture was created inside the GelMA microstructures via embedding. Figure 2 shows the experimental procedure used to fabricate cells embedded the GelMA microstructures. In the experiments, three different types of cells were used: rat liver cells (RLC-18), mouse fibroblast cells (NIH3T3), and mouse smooth muscle cells (SMCs) (Riken BioResource Research Center, Tsukuba, Japan). To fabricate microstructures, cells were mixed into the 10% GelMA + 0.5% photo initiator (PI) solution. Irgacure 2959 was used as the photo initiator (PI). The UV light was then shone through the 60× objective lens and the mask of the toroidal shape. The GelMA solution with cells was exposed to UV light for 10 s. The solution around the microstructures was then replaced in the culture medium (Dulbecco’s modified Eagle medium (DMEM) + 10% fetal bovine serum (FBS)). The cells around the GelMA microstructures were removed from the culture dish during solution replacement. The cells inside the GelMA microstructures were then cultured inside a 37 °C CO_2_ incubator.

To check cell viability of the cell culture inside the GelMA microstructures, a live/dead assay was conducted. Calcein acetoxymethyl ester (Calcein-AM) (Life Technologies, Thermo Fisher Scientific Japan, Tokyo, Japan) was mixed into the culture medium at 0.2 g/mL, and the cells were cultured for 15 min in the 5 % CO_2_ incubator. After 3 days of cell culture inside the GelMA, propidium iodide (Life Technologies, Thermo Fisher Scientific Japan, Tokyo, Japan) was used to check for dead cells. This was mixed into the culture medium at 2.5 g/mL, and cells were cultured for 5 min.

### 2.3. Cell Culture on GelMA Surface

In our experiment, the UV power source could output power at six different percentages: 3%, 6%, 12%, 25%, 50%, and 100%. To check the fabrication conditions, the UV power at the fabrication area of the microstructure was measured. The fabrication area was exposed to the UV light through the 60× objective lens.

To check the cell growth on the GelMA microstructures, a cell culture was conducted. In the experiment, rat liver cells (RLC-18) were used. First, toroidal microstructures were fabricated as follows. The lyophilized GelMA was dissolved in the culture medium (DMEM + 10% FBS) to make a concentration of 5% w/v GelMA solution. The PI powder was then dissolved in dimethyl sulfoxide (DMSO) solvent to obtain a concentration of 50% w/v as the stock solution. Before fabricating the GelMA structures, the experimental solution was prepared by mixing the 5% w/v GelMA solutions and 50% w/v PI solution. The PI concentration became 0.5% w/v in the experimental solution. A mask of toroidal shape was then used to fabricate the toroidal shapes of the microstructures.

**Figure 3:**
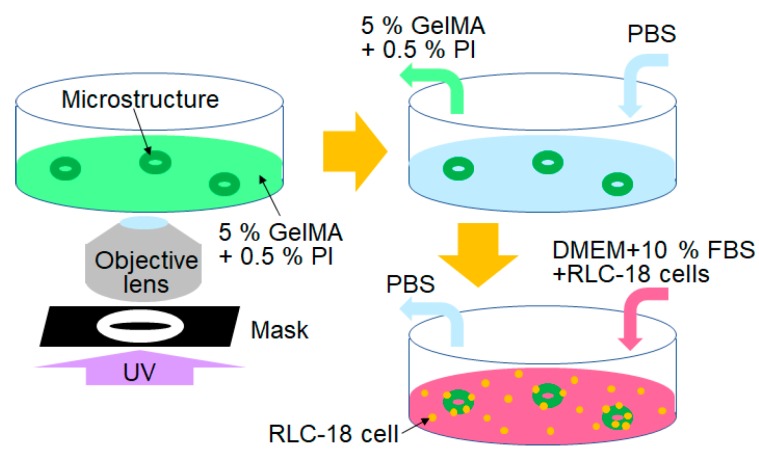
Experimental procedure of cell culture on GelMA microstructures.

Figure 3 shows the experimental procedure of cell culture on GelMA microstructures. First, we prepared 5% GelMA solution with 0.5% w/v PI solution placed inside an ultra-low attachment dish (corning). The ultra-low attachment dish was then used to prevent cell adhesion around the GelMA microstructures. The dish was then exposed to UV light for 10 s through the mask. After the solidification of GelMA, uncured GelMA solution was replaced with PBS, and then with a culture medium (DMEM + 10% FBS) and rat liver cells (RLC-18) to seed the GelMA microstructures. The cells on the GelMA microstructures were then cultured inside a 37 °C 5% CO_2_ incubator. In the experiments, four different UV exposure durations (20, 30, 45, and 60 s) were tested for fabrication of the GelMA microstructures.

## 3. Results

### 3.1. Preparation of Biodegradable Microstructures

**Figure 4:**
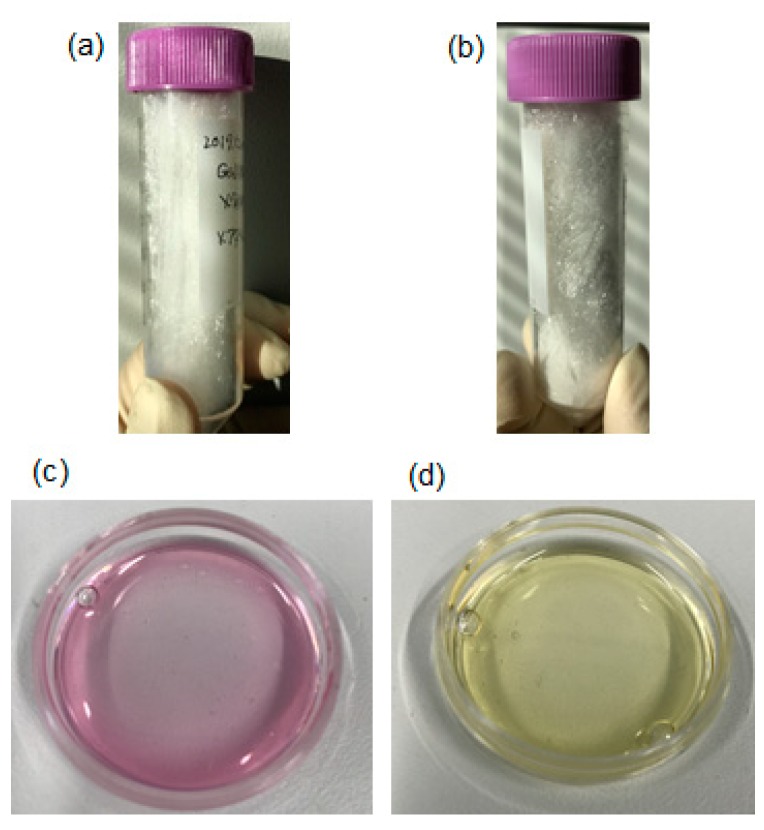
GelMA synthesis under different conditions: (**a**) lyophilized GelMA, dialyzed at 40 °C; (**b**) lyophilized GelMA, dialyzed at room temperature; (**c**) dissolved GelMA in a culture medium dialyzed at 40 °C; and (**d**) dissolved GelMA in a culture medium dialyzed at room temperature.

A photo-cross-linkable and biodegradable gelatin methacrylate (GelMA) hydrogel was synthesized. During GelMA synthesis, the dialysis temperature is an important parameter. Figure 4 shows the GelMA synthesized at different dialysis temperatures: 40 °C and room temperature (~20 °C). As shown in Figure 4a,b, there was no significant difference in their appearance after lyophilization. However, the acid levels differed. Figure 4c,d shows the color of the culture medium with phenolsulfonphthalein when the lyophilized GelMA was dissolved into the culture medium. The phenolsulfonphthalein changed its color depending on the pH. The color was red when the pH was higher than 8.0, but the color became yellow when the pH was lower than 6.6. Generally, cells need to be cultured in a culture medium at around pH 8 (red color conidtion of phenolsulfonphthalein-containing culture medium). When the GelMA was dialyzed at 40 °C, the dissolved GelMA culture medium became red. However, the dissolved GelMA culture medium became yellow (Figure 4d) and its pH was about 4 when the GelMA was dialyzed at room temperature. Notably, excess methacrylic acid in the GelMA was removed during dialysis so that the dialysis could be conducted at 40 °C. The viscosity of GelMA can be decreased by heating at 40 °C because the main material of GelMA is gelatin, and the dialysis process can be promoted by low viscosity of the GelMA. The dissolved GelMA culture medium after dialysis at 40 °C showed a red color (Figure 4c), and its pH was about 8. 

### 3.2. Cell Culture in GelMA Microstructures

In our method [22], cells were mixed in GelMA solution and encapsulated inside the GelMA microstructures by exposing them to patterned UV radiation. Such on-chip fabrication can be achieved by using patterned UV light through a mask. However, when the cells are encapsulated inside GelMA microstructures, only a few cells are stretched and grown [22]. In this study, we created cell cultures inside and on the GelMA microstructures to check the differences of cell growth.

**Figure 5:**
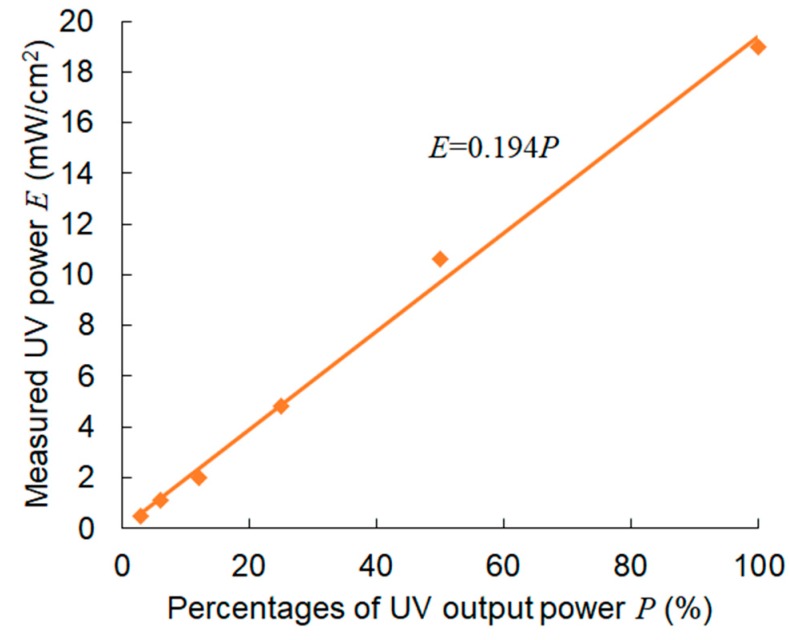
Measurement of UV power when a 60× objective lens was used.

To fabricate GelMA microstructures, UV power is important. Figure 5 shows the results of the UV power measurement. The UV power increased nearly proportionally with the output percentages. The relationship between UV output power, *P* (%), and the measured UV power, *E* (mW/cm^2^), in our experimental setup could be expressed using linear approximation.

*E* = 0.194*P*. (1)

When we fabricated the GelMA microstructures, UV exposure was set between 10 and 60 s, which corresponded to 190 and 1140 mJ/cm^2^ UV power, respectively. Thus, 10 s was the minimum duration to solidify the solution.

**Figure 6:**
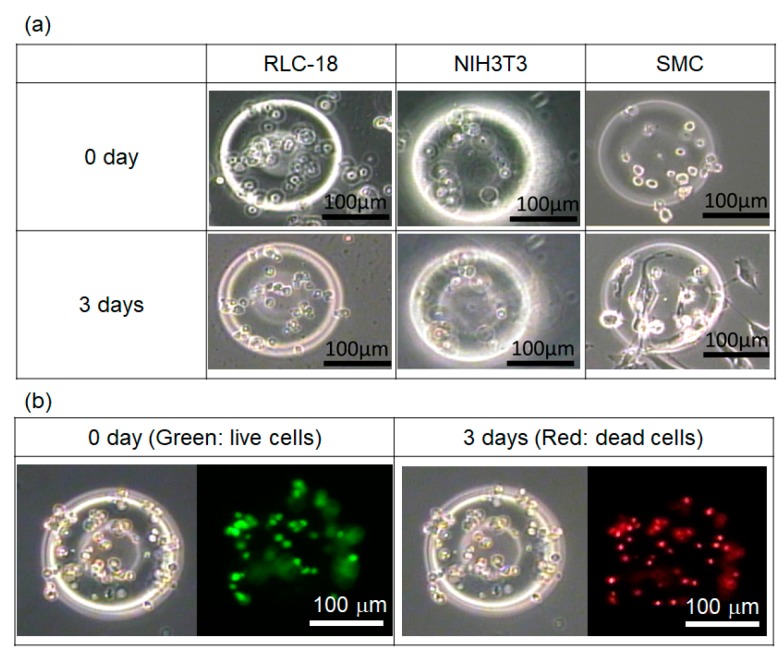
Cell culture in GelMA microstructures: (**a**) culture results using different cell types and (**b**) live/dead assay of RLC-18 cells in GelMA microstructure.

Figure 6a shows the experimental cell culture results from three cell types (i.e., rat liver cells RLS-18, mouse fibroblast NIH3T3, and mouse smooth muscle cells SMCs) after fabricating toroidal GelMA microstrucrtures using 10 s UV irradiation. In the case of RLC-18 and NIH3T3, cell shapes showed almost no change during the 3 days of cell culture, and cell growth was not observed. In the case of SMC, a few stretched cells were observed. However, most cells maintained a round shape, and the cell numbers did not increase. The results indicated that the stretched cells may not have been fully embedded inside the GelMA solution and were instead attached on the surface. However, the round cells were fully embedded in the GelMA microstructures and could not be well stretched.

A live/dead assay was conducted to check cell viability in the GelMA microstructures. This was accomplished by using fluorescent dyes, calcein-AM and propidium iodide. Calcein-AM stains only live cells green fluorescent, and propidium iodide stains only dead cells red fluorescent. Just after the fabrication of cell-embedded GelMA microstructures, calcein-AM was used to check for live cells. The experimental results are shown in Figure 6b. Many cells remained alive just after the fabrication of the GelMA microstructure. However, most cells were dead after 3 days of cell culture. The results indicated that the environment inside the GelMA was not suitable for cell growth in our experimental setup.

### 3.3. Cell Culture on GelMA Surface

To enhance cell growth, the fabrication procedure of the cell microstructure was changed. In the new fabrication procedure, the GelMA microstructures were fabricated first, without cells. Cells were seeded onto the fabricated GelMA microstructures. Thus, the cells could be attached and stretched on the surface of the GelMA microstructures.

**Figure 7:**
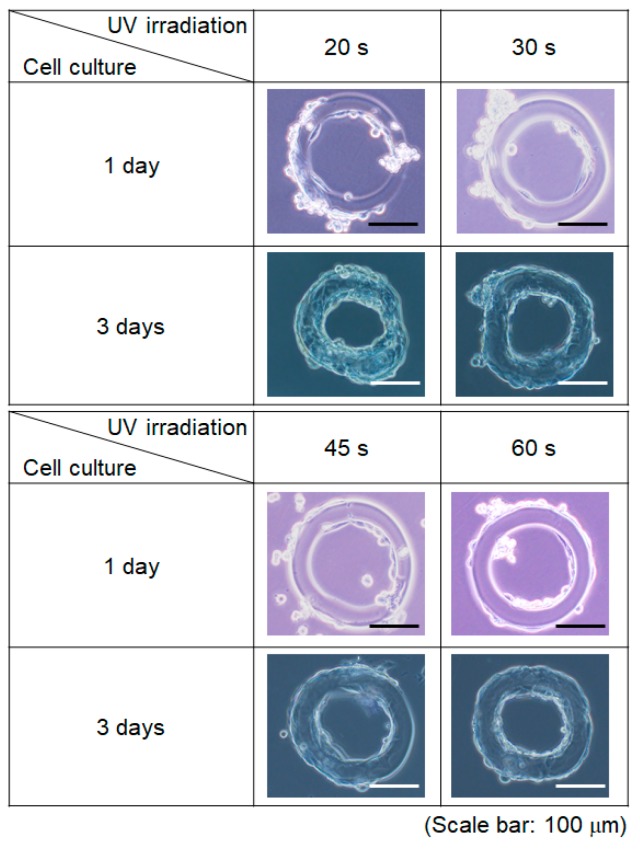
Cell culture on GelMA microstructure after 2 days of different UV exposures.

Figure 7 shows the results of culturing SMCs on the GelMA microstructures. After 1 day of cell culture, cells were attached onto the surface of GelMA microstructures, and some were stretched in all conditions. After 3 days, the cell numbers had increased, and the surfaces of GelMA microstructures were covered by cells. After 20 and 30 s UV irradiation, the GelMA microstructures were deformed because of the stretching force from the cells. However, the shape of the GelMA microstructures stayed round in cases of 45 and 60 s UV irradiation. The results indicated that the longer UV irradiation increased the mechanical strength of the GelMA, and that the microstructures therefore kept their shapes even when the cells covered the surface. 

**Figure 8:**
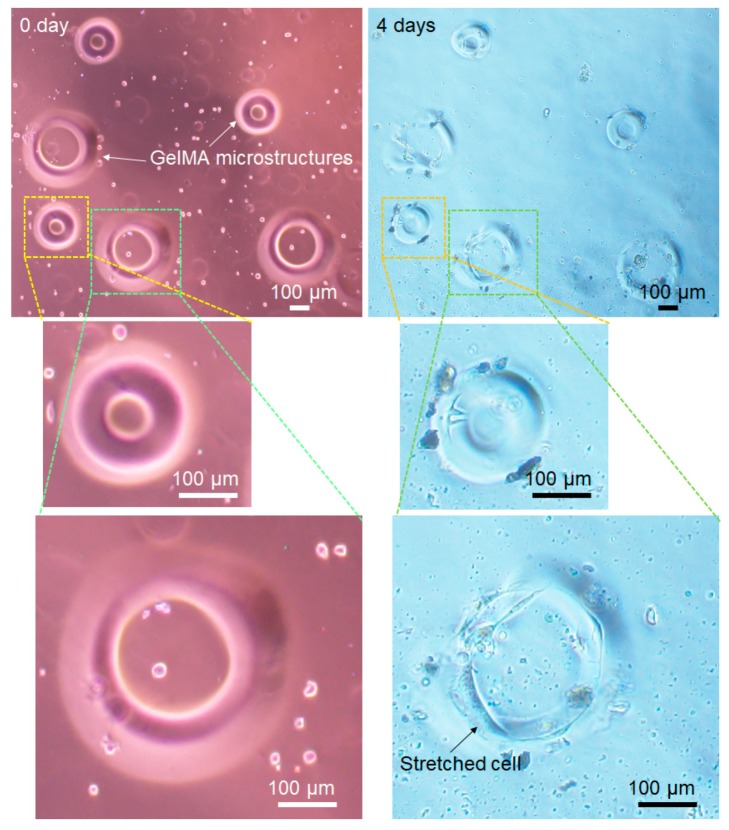
Degradation of GelMA during cell culture. Just after the fabrication (0 day), all structures were adhered to the substrate, showing a clear edge. Larger microstructures had larger number of cells on it and they showed deformation and degradation after 4 days, while smaller microstructures maintained a clear edge after 4 days.

The degradation of the GelMA microstructures was checked using RLC-18 cells. In the experiment, cell numbers were decreased to visualize the GelMA degradation. Figure 8 shows the experimental results. In this experiment, two different sizes of microstructures were fabricated by changing the mask size, with 170 and 280 m outer diameters. Just after the fabrication (0 day), all structures were adhered to the substrate, showing a clear edge. Some microstructures were deformed and degraded after 4 days. The speed of degradation appeared to be different according to the number of cells on the microstructure.

Smaller toroidal structures on the left side (magnified at the center in Figure 8) maintained a relatively clear edge compared with the larger toroidal microstructures of the smaller microstructure. The larger toroidal microstructure (magnified in the bottom of Figure 8) had larger number of cells on it. The results indicated that the cells on the GelMA microstructures could be cultured according to the GelMA patterns. Additionally, the GelMA could be dissolved by the cell culture because of its biodegradable character.

### 3.4. Movable GelMA Microstructures in a Microfluidic Device

**Figure 9:**
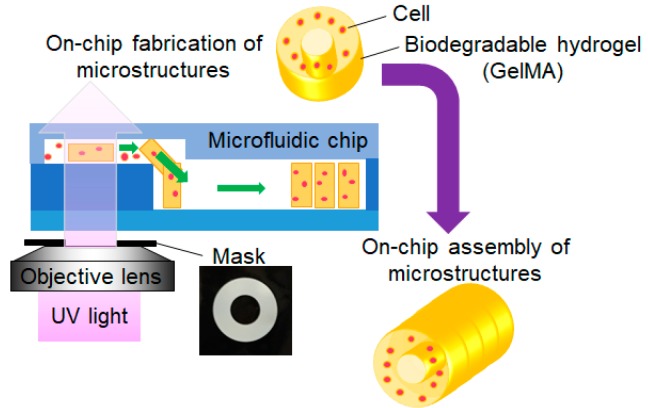
Fabrication procedure of tubular microstructures using on-chip fabrication and the assembly of toroidal cell-embedded microstructures.

In our method, 3D cell structures were fabricated using GelMA. Figure 9 shows the fabrication procedure of tubular cell structures using a photo-cross-linkable biodegradable hydrogel. In our method, 2D toroidal microstructures were used to assemble 3D tube shapes. The fabricated microstructures were movable when the water-repellent coating was applied to the surface of the microchannel [22]. The fabrication and assembly of microstructures could thus be conducted using the same microfluidic chip. The assembled GelMA can be degraded, resulting in only the cell structures remaining in the microfluidic chip.

**Figure 10:**
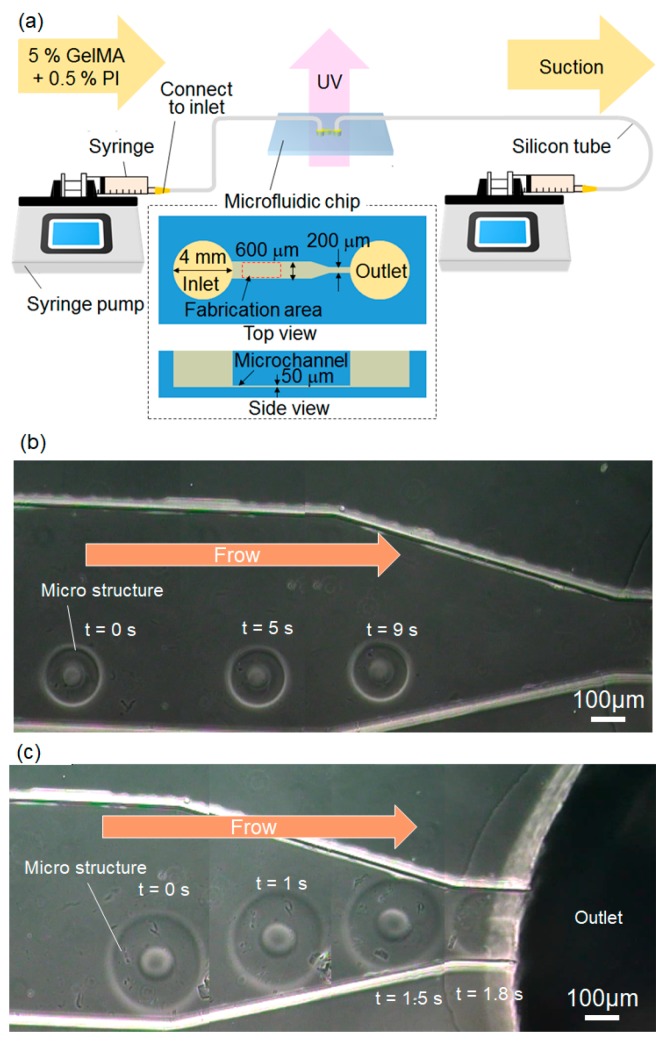
On-chip fabrication of movable GelMA microstructures: (**a**) experimental setup; (**b**,**c**) movable microstructures inside the microfluidic chip.

Figure 10 shows the fabrication of movable GelMA microstructures using a microfluidic chip. Figure 10a shows the experimental setup used to check the movability of GelMA microstructures. The inlet and outlet of the microfluidic chip were connected to syringe pumps via silicon tubes. The fabrication area was exposed to UV light via a mask and an objective lens. The microfluidic chip was made of polydimethylsiloxane (PDMS) and fabricated using a general soft-lithography process. The channel was coated by a water-repellent coating material to peel off the microstructures after fabrication [22]. In the microchannel, toroidal GelMA microstructures were fabricated using patterned UV light. After fabrication, the syringe pumps were turned on, and the flow in the microchannel generated enough force to peel off the fabricated microstructures. As shown in Figure 10b,c, two different sizes of GelMA microstructures were moved through the microchannel by controlling the flow rate of the syringe pumps. Thus, these types of movable microstructures can be used as building blocks for 3D assembly. By choosing toroidal shapes, the assembled microstructures can then be formed into tube shapes, as shown in Figure 8.

**Figure 11:**
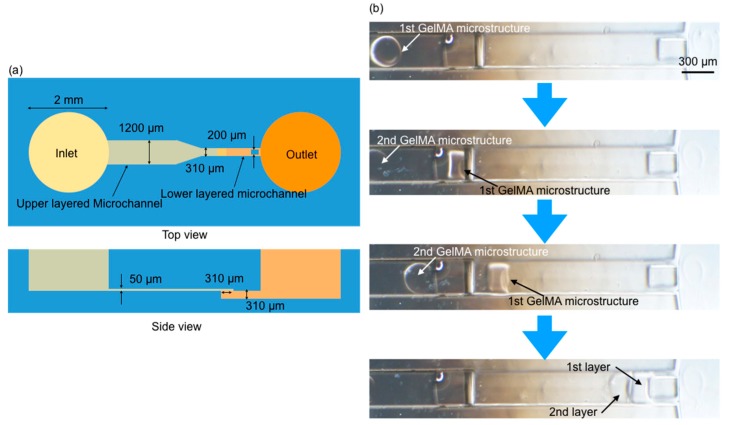
On-chip assembly of movable GelMA microstructures: (**a**) design of two-layered microfluidic device; (**b**) experimental results of two-layered assembly of GelMA microstructures.

Figure 11 shows the experimental result of self-assembly of GelMA microstructures inside the two-layered microfluidic device. In the experiment, a two-layered microchannel designed as shown in Figure 11a was used. The toroidal-shaped GelMA microstructures were flowed inside the upper layer first, and a syringe pump connected to the outlet of the microchannel was used to suck the solution inside the microchannel. The two GelMA microstructures were moved inside the channel, and they were rotated 90° at the connection between upper and bottom layer of the channel as shown in Figure 11b. They were finally stacked inside the bottom-layered channel. Hence, the experimental results showed that the GelMA microstructures could be used to construct tube-shaped cell structures using our self-assembly method inside a microchannel.

## 4. Conclusions

We created a cell culture with GelMA microstructures. The biodegradable hydrogel GelMA was employed to fabricate cell microstructures. The synthesis procedure of GelMA was changed to achieve suitable conditions. The temperature of dialysis was important for removing excess methacrylic acid inside the GelMA. We experimentally confirmed that the cell-embedded conditions were not suitable for cell culture.

The cells were then seeded on the surface of GelMA microstructures to enhance cell growth. Rat liver cells were stretched on the GelMA microstructures, and nearly the entire surface of the GelMA was covered with cells after 3 days. Partial degradation of the GelMA microstructures was observed after 4 days of cell culture. The degradation speed depended on the cell numbers attached to the surface of the GelMA. The pattern of GelMA microstructures can be transferred to the shape of cell structures using our proposed method. The fabricated toroidal microstructures were successfully manipulated in the two-layered microfluidic chip to be assembled into a tube shape. The results indicate that the GelMA microstructures can be used as a building blocks of 3D in vitro tissue-like cell structures.

Recently, 3D bioprinting technology has become one of the most promising techniques for the construction of 3D cellular structures. However, bioprinting requires a longer time to construct larger structures, since they have to be constructed from 0D (dot) to 3D (volume). Our method can be used to assemble 2D microstructures using a self-assembly process. A tube shape fabricated inside a microfluidic device using the self-assembly process can be used as a part of vascular networks. Vascular-like channel structures are necessary for constructing large 3D cell structures without necrosis. Generally, capillary networks (5–10 μm diameter) can be constructed automatically inside cell structures via the self-organization of cells when the vascular endothelial cells are properly mixed and cultured. However, artificial blood vessels larger than 6 mm have already been developed and clinically used. Thus, relatively larger blood vessels of several hundred micrometers have not been prepared. The method demonstrated in this paper can be used to fabricate tube shapes with the diameters required to construct relatively large 3D cell structures. Thus, the patterning of different cells in one structure is possible using our method [22].
